# Prognostic Significance and Clinical Implications of the New FIGO 2023 Staging System in Patients with Endometrial Cancer

**DOI:** 10.3390/jcm15166326

**Published:** 2026-08-16

**Authors:** Hakan Özçelik, Nazan Demir, Melisa Eryaşar, Oğuz Çelebi, Erdem Sünger, Burçin Çakan Demirel, Jamshid Hamdard, Özgür Açıkgöz, Burak Giray, Doğan Vatansever, İsa Aykut Özdemir, Volkan Ülker, Çağatay Taşkıran, Fatih Selcukbiricik, Ahmet Bilici

**Affiliations:** 1 Department of Medical Oncology, Faculty of Medicine, Istanbul Medipol University, Istanbul 34214, Turkey; erdemsunger@gmail.com (E.S.); burcin.cakandemirel@gmail.com (B.Ç.D.); jamshidhamdard@hotmail.com (J.H.); ozgur_acikgoz@yahoo.com (Ö.A.); ahmetknower@yahoo.com (A.B.); 2 Department of Medical Oncology, Koc University Hospital, Istanbul 34010, Turkey; ndemir@kuh.ku.edu.tr (N.D.); fselcukbiricik@kuh.edu.tr (F.S.); 3 Faculty of Medicine, Istanbul Medipol University, Istanbul 34214, Turkey; melisaery2002@gmail.com; 4 Department of Internal Medicine, Koc University Hospital, Istanbul 34010, Turkey; celebioguz59@gmail.com; 5 Department of Gynecologic Oncology, Koc University Hospital, Istanbul 34010, Turkey; burakgiray@gmail.com (B.G.); drdvatansever@gmail.com (D.V.); cagataytaskiran@yahoo.com (Ç.T.); 6 Department of Gynecologic Oncology, Faculty of Medicine, Istanbul Medipol University, Istanbul 34214, Turkey; isaaykutozdemir@hotmail.com (İ.A.Ö.); drvolkanulker@yahoo.com (V.Ü.)

**Keywords:** endometrial cancer, FIGO 2023, lymphovascular space invasion, molecular classification, overall survival, prognosis, progression-free survival, risk stratification, stage migration, surgical margin

## Abstract

**Background/Objectives:** The 2023 revision of the International Federation of Gynecology and Obstetrics (FIGO) staging system redefines endometrial cancer staging by integrating histopathological and molecular parameters into conventional anatomic assessment. We aimed to quantify stage migration following reclassification from FIGO 2009 to FIGO 2023, to evaluate its impact on survival, and to identify independent prognostic determinants within this restaged cohort. **Methods:** In this two-center retrospective cohort study, 291 patients with histologically confirmed endometrial cancer were restaged according to both FIGO 2009 and FIGO 2023 systems. Progression-free survival (PFS) and overall survival (OS) were assessed using the Kaplan–Meier method, and independent prognostic determinants were identified through multivariable Cox proportional hazards modeling. **Results:** Application of FIGO 2023 resulted in stage migration in 32.0% of patients, with 30.6% upstaged and 1.4% downstaged. Upstaged patients demonstrated significantly worse PFS and OS compared with downstaged patients and patients without stage change (*p* < 0.001 for both). Notably, among patients initially classified as FIGO 2009 stage I, 38% were reassigned to a higher stage and exhibited a marked survival disadvantage (*p* < 0.001). In multivariable analysis, advanced age and positive surgical margins independently predicted both poorer PFS and OS, while lymphovascular space invasion (LVSI) independently predicted inferior PFS. The association with positive surgical margins, however, likely reflected concurrent advanced-stage disease. **Conclusions:** The FIGO 2023 staging system results in substantial stage redistribution in endometrial cancer and provides clearer survival discrimination, particularly by identifying previously unrecognized high-risk subgroups within early-stage disease. The significant survival disadvantage observed among upstaged patients indicates that the revised system may more accurately reflect biologically driven risk stratification. However, stage migration was not independently associated with survival after adjustment for its constituent pathological factors.

## 1. Introduction

Endometrial cancer is a malignant epithelial tumor of the uterine corpus and is the most common gynecologic malignancy in the United States [1] and the fourth most common malignancy among women [2]. Although most patients are diagnosed at an early stage, high-grade and other high-risk histological subtypes are associated with substantially higher recurrence rates and considerably poorer survival [3]. Therefore, accurate identification of prognostic factors is of critical importance in determining appropriate treatment strategies in endometrial carcinoma.

In patients diagnosed with endometrial cancer, prognosis largely depends on the stage of the disease [2]. Even when immunohistochemical evaluation is used, variability may still be observed among expert pathologists in determining clinicopathological prognostic factors. This variability is most pronounced in the assignment of tumor grade and histological subtype—particularly in high-grade carcinomas—and in the evaluation of lymphovascular space invasion (LVSI), which are among the least reproducible parameters in endometrial cancer pathology [4,5]. The new World Health Organization (WHO) classification published in 2020 aimed to reduce these inconsistencies by integrating morphological, immunohistochemical, and molecular diagnostic features [6]. This heterogeneity has led to both overtreatment and undertreatment in thousands of cases across cancer centers worldwide [4,5]. In 2013, The Cancer Genome Atlas (TCGA) Research Network defined a molecular classification system for endometrial cancer to enable more accurate prognostic prediction and appropriate treatment selection. Four molecular subgroups were identified: DNA polymerase epsilon–mutated (POLEmut) subtype, mismatch repair-deficient subtype (dMMR), no specific molecular profile subtype (NSMP), and p53 abnormal (p53abn) subtype [6].

Notably, the POLE ultramutated subtype of endometrial cancer has been shown to have an excellent prognosis [7]. dMMR tumors are frequently associated with adverse pathological features such as higher grade and LVSI, yet paradoxically may show favorable clinical outcomes [8]. The p53abn endometrial cancer subtype is associated with the poorest prognosis and appears to have the greatest potential to benefit from aggressive adjuvant therapy [9]. Finally, although NSMP endometrial carcinomas are generally classified within the intermediate-risk group, more detailed subclassifications are needed to enable a more individualized approach for these tumors [10].

These four molecular subgroups were integrated with the previous classification system in the newly published European Society of Gynaecological Oncology (ESGO), European Society for Radiotherapy and Oncology (ESTRO), and European Society of Pathology (ESP) guidelines to establish the current molecular classification approach for the optimal management of patients with endometrial cancer [11]. In 2023, the International Federation of Gynecology and Obstetrics (FIGO) introduced the first revision of the endometrial cancer staging system since 2009 [2]. The FIGO 2023 staging system includes several important changes [12]. First, due to its prognostic significance in early-stage disease, histological type has been incorporated into the staging criteria as a binary classification (non-aggressive and aggressive) [13]. Second, LVSI, an important prognostic parameter in endometrial cancer [14,15] has been included in the staging system. Third, certain stages have been subdivided into more detailed subgroups according to anatomical disease distribution, and the number of substages has been increased from 9 to 19. Finally, the staging system has been expanded to incorporate four molecular biomarker statuses. While the FIGO 2009 staging system is based on the extent of surgical and anatomical tumor invasion and the presence of metastasis, the FIGO 2023 staging system integrates well-defined prognostic factors such as histological type, tumor grade, and LVSI with molecular classification. Particularly in Stage I and II disease, molecular classification may lead to stage modification depending on p53abn or POLEmut status, resulting in upstaging of the disease to IICmp53abn or downstaging to IAmPOLEmut, respectively [12].

These changes have led to notable differences in stage distribution compared with the FIGO 2009 staging system. Therefore, this study aimed to investigate stage migration between the FIGO 2009 and FIGO 2023 staging systems and to evaluate the clinical and prognostic impact of the updated classification in patients with endometrial cancer. To our knowledge, this is one of the few two-center, real-world cohort studies to systematically restage every patient under both the FIGO 2009 and FIGO 2023 systems and to jointly report stage migration, molecular characterization, and independent prognostic determinants; its relatively high rate of molecular assessment and its explicit focus on the previously unrecognized high-risk subgroup hidden within FIGO 2009 Stage I disease distinguish it from earlier reports.

## 2. Materials and Methods

### 2.1. Data Source/Study Characteristics

The medical records of patients with endometrial cancer treated at the Medical Oncology Departments of Istanbul Medipol University and Koc University were retrospectively reviewed; the medical records were examined during the study period (November 2025–February 2026). Patients diagnosed between December 2012 and February 2025 (and surgically treated between January 2013 and June 2025) were included. The study protocol was approved by the Ethics Committee of Medipol University (Istanbul, Turkey) on 17 September 2025 (reference number: E-10840098-202.3.02-6283). Written informed consent was obtained from all patients or their legal representatives.

### 2.2. Patients

The study included patients aged ≥18 years with histologically confirmed endometrial cancer. Of those who met the eligibility criteria, 291 patients were included in the final analysis. Patients with synchronous malignancies or missing essential clinical or pathological data were excluded. Demographic characteristics, tumor features, treatment information, and follow-up data were retrospectively obtained from the hospital data systems.

### 2.3. Data Collection

Data extracted from electronic medical records included age and date of diagnosis, as well as surgical information including operation status, date of surgery, and type of surgery. Regarding pathological evaluation, pathological tumor–node–metastasis (pTNM) stage, LVSI, depth of myometrial invasion, total number of lymph nodes removed, number of metastatic lymph nodes, tumor diameter (cm), surgical margin status (positive or negative), histological subtype, histological type (aggressive or non-aggressive), tumor grade, and molecular classification were recorded. In addition, FIGO 2009 stage, FIGO 2023 stage, estrogen receptor (ER) and progesterone receptor (PR) status, and administered treatments (including neoadjuvant chemotherapy, adjuvant chemotherapy, radiotherapy, and brachytherapy) were documented. Time-dependent variables included date of diagnosis, date of progression, date of last follow-up, and/or date of death. Molecular characterization was performed on formalin-fixed, paraffin-embedded tissue. Universal immunohistochemical screening for mismatch repair (MMR) proteins and for p53 expression was institutional policy at both participating centers; because of the retrospective design, however, interpretable results could not be retrieved for the entire cohort. Staining was performed on a BenchMark ULTRA automated platform using the following prediluted primary antibodies (Ventana Medical Systems/Roche Diagnostics, Tucson, AZ, USA): MLH1 (clone M1), PMS2 (clone A16-4), MSH2 (clone G219-1129), MSH6 (clone SP93), and p53 (clone BP53-11). The same platform and antibody clones were used at both centers. MMR deficiency was defined as loss of nuclear expression of one or more MMR proteins, and abnormal p53 as diffuse strong overexpression, complete absence, or unequivocal cytoplasmic staining. MLH1 promoter hypermethylation analysis was not performed. POLE exonuclease-domain mutation testing was not available in-house; when POLE status was required, the most representative tumor block was referred to an external accredited molecular pathology center for next-generation sequencing (NGS), targeting the POLE exonuclease domain (exons 9–14). As this was a retrospective study, POLE sequencing was not performed according to a study-defined selection rule; all eligible patients were included, and POLE status was available only in those in whom sequencing had been requested as part of routine clinical care. Patients without a definitive molecular result were recorded as unknown.

LVSI was categorized as absent, focal, or substantial according to the most recent WHO classification. Focal LVSI was defined as the presence of lymphovascular invasion in one to four lymphovascular spaces around the tumor, whereas substantial LVSI was defined as the presence of tumor cells in five or more lymphovascular spaces [16]. The depth of myometrial invasion was evaluated in three categories based on the percentage of total myometrial thickness infiltrated by carcinoma: no invasion, <50% invasion, and ≥50% invasion [17,18]. Histological types were classified as follows: endometrioid carcinoma, serous carcinoma, clear cell carcinoma, mixed carcinoma, undifferentiated carcinoma, carcinosarcoma, mesonephric-like carcinoma, and gastrointestinal-type mucinous carcinoma. LVSI and histopathological parameters were evaluated by dedicated gynecologic pathologists at each participating center according to the current WHO criteria; a formal central pathology review and quantitative interobserver-reliability assessment (e.g., kappa statistics) were not performed, which is acknowledged as a limitation. Non-aggressive histological types consisted of low-grade (grade 1–2) endometrioid carcinomas, whereas all other histological types (i.e., serous, clear cell, mixed, undifferentiated, carcinosarcoma, mesonephric-like, and gastrointestinal-type mucinous carcinomas) and high-grade (grade 3) endometrioid carcinomas were considered aggressive [13]. Cases harboring pathogenic POLE exonuclease domain mutations were classified as POLEmut regardless of MMR or p53 status, while POLE wild-type tumors with MMR deficiency were classified as dMMR independent of p53 expression [9]. Accordingly, tumors fulfilling the criteria for more than one molecular subtype (multiple-classifier tumors) were assigned according to this predefined hierarchy (POLEmut > dMMR > p53abn), in line with the WHO and ESGO/ESTRO/ESP algorithm underlying FIGO 2023.

All patients were initially staged according to the FIGO 2009 staging system [19], and subsequently reclassified according to the FIGO 2023 staging system [12]. Both FIGO staging systems are presented in Supplementary Table S1 and Table S2, respectively. Reclassification to a higher or lower stage group was defined as “upstaging” or “downstaging,” respectively.

Progression-free survival (PFS) and overall survival (OS) were compared among patients who were upstaged, downstaged, or remained at the same stage according to the FIGO 2023 staging system. In addition, patients classified as Stage I according to FIGO 2009 were analyzed in terms of PFS and OS between those with stage increase and those without stage change. PFS was defined as the time from surgery to recurrence or last follow-up, and OS as the time from surgery to death or last follow-up.

### 2.4. Statistical Analysis

All data obtained from participating centers were pooled for statistical evaluation. Descriptive statistics were used to summarize the study variables, including frequencies, percentages, means, standard deviations, medians, and ranges. Survival outcomes were analyzed using the Kaplan–Meier method, and differences between groups were assessed with the log-rank test. To identify factors associated with PFS and OS, univariate and multivariate analyses were performed using the Cox proportional hazards regression model. Variables with statistical significance in univariate testing were subsequently entered into the multivariate model through a stepwise selection approach. To avoid collinearity between the composite stage-migration variable and its constituent pathological components, stage migration was not entered together with these variables in the final multivariable models. Accordingly, because the FIGO 2023 stage group is likewise derived from these constituent pathological components (LVSI, histological subtype, molecular class, and depth of myometrial invasion), it was evaluated in a separate multivariable model as a categorical variable (I–IV), adjusting only for age and surgical margin status. Results were expressed as hazard ratios (HRs) with corresponding 95% confidence intervals (CIs). Missing values for individual covariates were retained as a separate ‘unknown’ category and were not imputed; all patients were retained in the models, with the missing observations forming the ‘unknown’ category for that variable (LVSI was unknown in 17 patients, surgical margin status in 22, ER and PR status in 86 and 116, respectively, and molecular subtype in 71). The proportional-hazards assumption was assessed using scaled Schoenfeld residuals and was satisfied for all models (global *p* = 0.16 for PFS and *p* = 0.32 for OS). In a sensitivity analysis additionally adjusting for adjuvant chemotherapy and radiotherapy, the FIGO 2023 stage group remained independently associated with both PFS and OS. All statistical analyses were carried out using Statistical Package for the Social Sciences (SPSS) Statistics software, version 27.0 (IBM Corp., Armonk, NY, USA). A two-sided *p*-value of less than 0.05 was considered indicative of statistical significance.

## 3. Results

A total of 291 women with endometrial cancer were included in this retrospective cohort. The patient selection process, molecular data availability, and the handling of missing data are summarized in the study flow diagram (Supplementary Figure S1). The median age at diagnosis was 61 years (interquartile range [IQR]: 54–68). The median number of lymph nodes removed was 20 (IQR: 8–36.75). The median tumor diameter was 3.5 cm (IQR: 2–5). Of the patients, 56% (*n* = 163) were aged ≥60 years. Nearly all patients underwent surgery (99.7%; *n* = 290). Regarding surgical approach, 51% (*n* = 148) underwent laparoscopic surgery, 45.5% (*n* = 132) underwent laparotomy, and 2.1% (*n* = 6) underwent robotic surgery.

Regarding pathological T stage, pT1A was the most common finding (50.9%; *n* = 148), followed by pT1B (23.7%; *n* = 69) and pT3A (11.3%; *n* = 33). Regarding lymph node assessment, 77% (*n* = 224) of patients were classified as pN0, 9.6% (*n* = 28) as pN1, and 12% (*n* = 35) as pN2. Distant metastasis was detected in 11.3% (*n* = 33) of patients. LVSI was present in 35.4% of patients, of which 5.2% (*n* = 15) was focal and 30.2% (*n* = 88) was substantial. In terms of depth of myometrial invasion, 41.9% (*n* = 122) had <50% invasion, 38.8% (*n* = 113) had ≥50% invasion, and 14.8% (*n* = 43) had no evidence of myometrial invasion.

According to histological classification, 42.3% (*n* = 123) of cases were categorized as aggressive and 57.7% (*n* = 168) as non-aggressive. When histological subtypes were evaluated, the most common subtype was endometrioid carcinoma (69.1%; *n* = 201), followed by serous carcinoma (19.6%; *n* = 57) and carcinosarcoma (4.5%; *n* = 13). Positive surgical margins were identified in 4.5% (*n* = 13) of patients. According to molecular classification, 27.8% (*n* = 81) were dMMR, 26.1% (*n* = 76) p53abn, and 21.3% (*n* = 62) NSMP, while POLEmut was detected in only one patient (0.3%). MMR immunohistochemistry was available in 229 patients (78.7%) and p53 immunohistochemistry in 237 (81.4%). POLE sequencing was performed in 68 patients (23.4%) and was positive in one (1.5%); all 62 NSMP tumors had been sequenced and were POLE wild-type.

Neoadjuvant chemotherapy was administered to 1.7% (*n* = 5) of patients, and 46% (*n* = 134) received adjuvant chemotherapy. Brachytherapy was performed in 38.8% (*n* = 113) of patients, and external beam radiotherapy was administered in 37.5% (*n* = 109). According to hormone receptor status, ER positivity was detected in 60.5% (*n* = 176) and PR positivity in 49.1% (*n* = 143) of patients. During follow-up, disease progression occurred in 23% (*n* = 67) of patients. The demographic and clinical characteristics of the patients are summarized in Supplementary Table S3.

The median follow-up was 29.2 months (95% CI: 24.6–31.3), estimated using the reverse Kaplan–Meier method. Neither median PFS nor median OS was reached in the entire cohort, owing to the limited number of events (67 progression events [23.0%] and 35 deaths [12.0%]). Because fewer than half of the patients experienced an event, restricted mean survival times are reported: the mean OS was 108.8 months (95% CI, 100.0–117.7) and the mean PFS was 89.1 months (95% CI, 78.0–100.2). The mean observation time was 31.7 months (standard deviation, 23.6). Stage increases were most frequently observed in the FIGO 2009 Stage IA and IB groups. In the Stage IA group, 4 patients were reclassified to Stage IC, 5 to Stage IIB, 24 to Stage IIC, and 12 to the IICmp53abn subgroup. In the Stage IB group, 6 patients were reclassified to Stage IIB, 18 to Stage IIC, and 1 to the IICmp53abn subgroup. In addition, 18 patients classified as Stage IVB according to FIGO 2009 were reclassified as Stage IVC under the FIGO 2023 system.

Stage decreases were observed in four patients; one patient was reclassified from Stage II to the IAmPOLEmut subgroup based on molecular staging, while the remaining three patients were downstaged from Stage IIIA to Stage IA3. Overall, 30.6% (*n* = 89) of patients were upstaged and 1.4% (*n* = 4) were downstaged under the FIGO 2023 system. The distribution of patients according to the FIGO 2009 and FIGO 2023 staging systems, as well as cases with stage increase (↑) and stage decrease (↓), are presented in Table 1.

**Figure 1:**
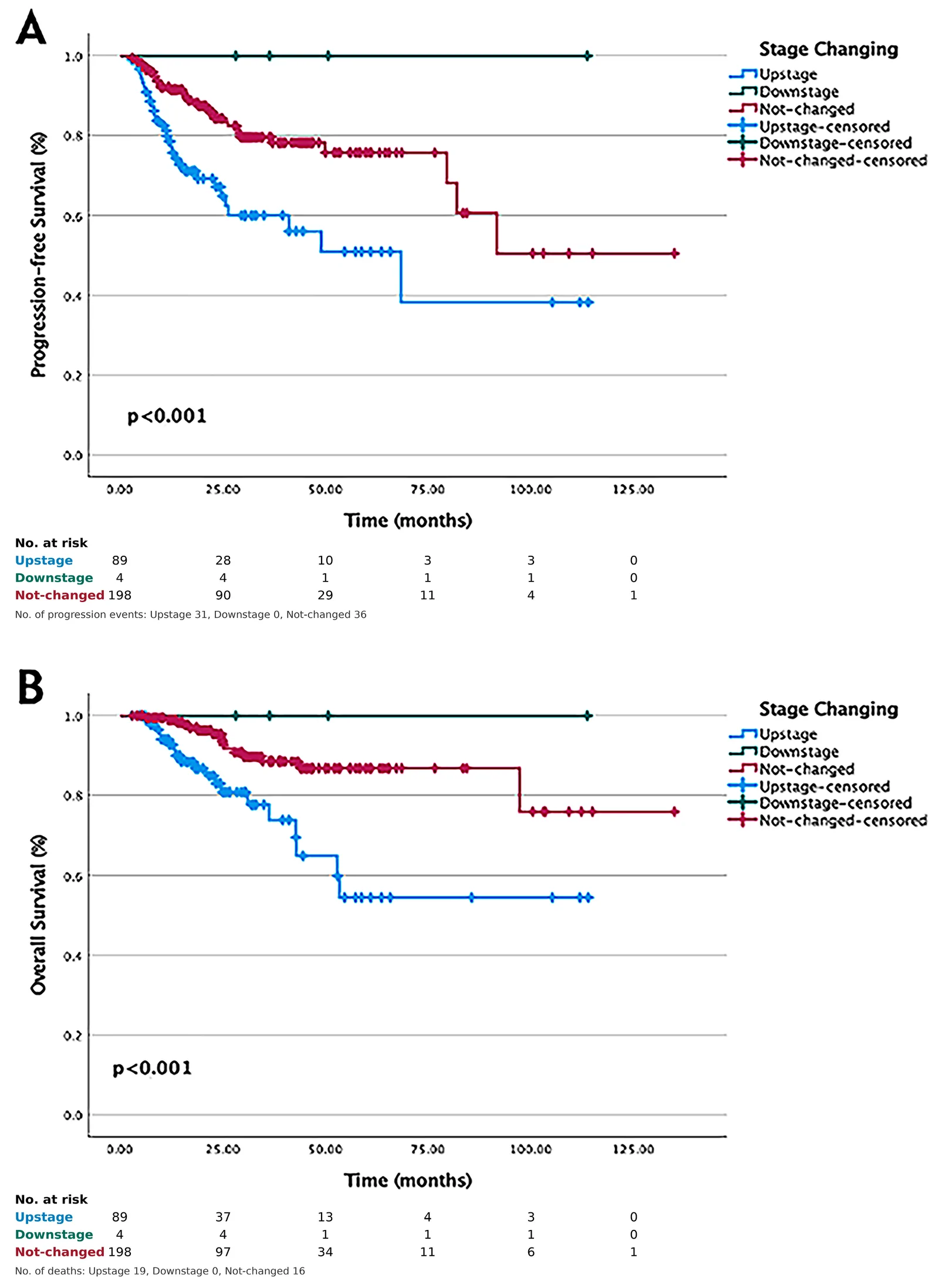
Kaplan–Meier survival curves according to stage migration from FIGO 2009 to FIGO 2023. (**A**) Progression-free survival and (**B**) overall survival stratified by upstaging, downstaging, and no stage change. Upstaged patients demonstrated significantly inferior outcomes compared with other groups (log-rank *p* < 0.001 for both). The numbers of patients at risk and the number of events per group are shown beneath each panel.

**Figure 2:**
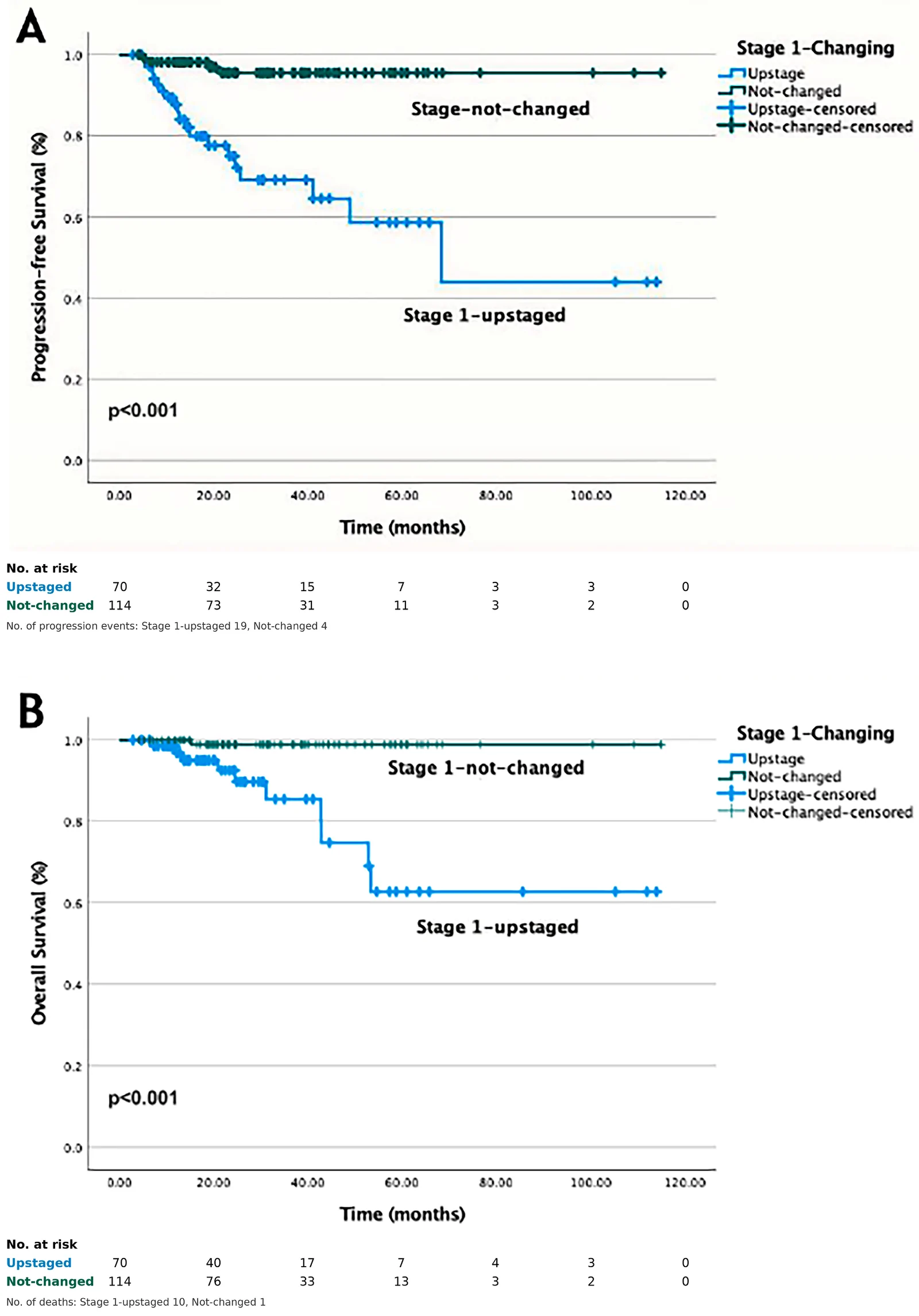
Kaplan–Meier survival curves in FIGO 2009 Stage I patients stratified by stage change after FIGO 2023 reclassification. (**A**) Progression-free survival and (**B**) overall survival comparing upstaged versus unchanged patients. Upstaged patients showed significantly inferior outcomes (log-rank *p* < 0.001 for both). The numbers of patients at risk and the number of events per group are shown beneath each panel.

In survival analyses based on stage change from FIGO 2009 to FIGO 2023 (upstaging, downstaging, and no stage change), upstaged patients had significantly shorter PFS and OS compared with the other groups (log-rank *p* < 0.001; Figure 1). Among the 184 patients classified as FIGO 2009 Stage I, 70 (38%) were upstaged after reclassification. Upstaged patients had significantly shorter PFS and OS compared with those without stage change (log-rank *p* < 0.001 for both; Figure 2).

In univariate analysis, several variables were associated with PFS including age, stage migration, LVSI, depth of myometrial invasion, histological characteristics, surgical margin status, molecular subtype and hormone receptor status (Table 2). In the multivariate Cox regression analysis, age category was independently associated with PFS (HR = 2.37; 95% CI: 1.31–4.29; *p* = 0.004). LVSI was also independently associated with PFS (HR = 1.45; 95% CI: 1.06–1.99; *p* = 0.022). Positive surgical margins were identified as an independent adverse prognostic factor for PFS (HR = 2.27; 95% CI: 1.58–3.27; *p* = 0.001) (Table 2).

In the univariate analysis for OS, several variables were significantly associated with survival, including age, stage migration, LVSI, depth of myometrial invasion, histological characteristics, surgical margin status, molecular subtype and hormone receptor status (Table 3). In the multivariate Cox regression analysis, age category was identified as an independent prognostic factor for OS (HR = 3.70; 95% CI: 1.31–10.46; *p* = 0.014). In addition, positive surgical margins were found to be an independent factor adversely affecting OS (HR = 2.56; 95% CI: 1.48–4.42; *p* = 0.001) (Table 3). When FIGO 2023 stage group (I–IV) was entered as a categorical variable in a separate multivariable Cox model (reference, stage group I), higher stage group was associated with significantly poorer PFS and OS relative to stage I (Supplementary Table S4).

## 4. Discussion

In this two-center retrospective study, the prognostic value and clinical implications of the FIGO 2023 staging system were evaluated in 291 patients with endometrial cancer. Reclassification according to FIGO 2023 resulted in substantial stage migration in 32% of patients, and stage increase was significantly associated with worse PFS and OS. Stage migration was most frequently observed among patients initially classified as FIGO 2009 Stage IA–IB. A considerable proportion of these patients were reassigned to higher substages or molecularly defined categories (e.g., IICmp53abn). These findings suggest that FIGO 2023 is capable of revealing a previously unrecognized “hidden high-risk” population among patients who were formerly categorized as low risk under the FIGO 2009 system. In addition, LVSI was identified as an independent adverse prognostic factor for PFS, while age and positive surgical margins were independently associated with both PFS and OS. Downstaging was observed in a small subset of patients, and their survival outcomes were comparable to those observed in low-stage disease.

The FIGO 2023 revision was designed to move beyond purely anatomical staging by incorporating histological characteristics, LVSI, and molecular biomarkers in order to improve prognostic accuracy [20,21]. Compared with the FIGO 2009 system, which relied predominantly on surgical and anatomical findings [19], FIGO 2023 aims to better capture tumor biological aggressiveness and refine risk stratification, particularly in early-stage disease [12,20,21]. In our study, stage increase was most frequently observed in the FIGO 2009 Stage IA–IB group, suggesting that the revised system is able to reveal a biologically aggressive subgroup that may have previously remained unrecognized. This observation is consistent with the integration of histological subtype, LVSI, and molecular biomarkers into the FIGO 2023 staging criteria [12]. In a large-scale analysis published in 2024, Matsuo et al. demonstrated that the prognostic performance of the FIGO 2023 system was significantly superior to that of FIGO 2009, particularly showing more distinct survival separation in early-stage disease [2]. Similarly, in an international pooled analysis from three ESGO-accredited centers, Schwameis et al. demonstrated that FIGO 2023 more clearly distinguished survival heterogeneity between stages and reshaped risk distribution. In early-stage disease, the new substages further increased prognostic precision and identified treatment-relevant subgroups [22]. Gravbrot et al., in their 2024 study, emphasized that the new system more accurately predicts survival outcomes by jointly considering anatomical involvement and histology [23]. Consistent with these findings, the significantly poorer PFS and OS observed in upstaged patients in our cohort (log-rank *p* < 0.001) are in line with the literature supporting the prognostic accuracy of FIGO 2023.

The significantly shorter PFS and OS observed in the 70 (38%) patients who were initially classified as Stage I and subsequently upstaged according to the new staging system suggest that some patients previously considered low-stage under the FIGO 2009 classification may be reclassified into higher-risk groups and may require different adjuvant treatment strategies. ESGO/ESTRO/ESP guidelines emphasize the importance of histology, grade, LVSI, and molecular features in risk stratification and treatment planning [11]. Similarly, reviews that integrate FIGO stage with biomarker and molecular classification in risk stratification indicate that this integration may strengthen clinical decision-making [10]. Therefore, the observed survival disadvantage in early-stage patients who were upstaged suggests that FIGO 2023 provides a staging approach more aligned with a “risk-adapted treatment” strategy [11,12].

The identification of LVSI as an independent adverse prognostic factor for PFS represents another important finding of our study. Previous studies, including pooled analyses of the PORTEC-1 and PORTEC-2 trials, have demonstrated the strong prognostic value of LVSI, particularly showing that substantial LVSI increases the risk of recurrence [14]. Furthermore, the depth and extent of LVSI have been reported to be associated with lymph node metastasis and recurrence patterns [15]. The persistence of LVSI as an independent factor in our multivariate analysis supports its inclusion in the FIGO 2023 staging system [12].

One of the key innovations of FIGO 2023 is the integration of molecular biomarkers into the staging system [12]. Molecular classification improves prognostic prediction in endometrial cancer [24]. Evidence from PORTEC-3 and NRG Oncology data has demonstrated the prognostic significance of p53abn and dMMR status [8,9]. Increasing evidence also suggests that the NSMP group represents a heterogeneous intermediate-risk category requiring further subclassification [10]. In our cohort, the prominent role of the p53abn subgroup in stage increases and survival separation underscores the clinical relevance of molecular-based staging in FIGO 2023, consistent with the risk-adapted approach recommended by ESGO/ESTRO/ESP guidelines [11].

In the multivariate analysis, age emerged as an independent prognostic factor for both PFS and OS. Consistent with our findings, a recent pooled analysis of the PORTEC-1, PORTEC-2, and PORTEC-3 trials demonstrated that advanced age is associated with more aggressive tumor characteristics and independently linked to poorer oncological outcomes in patients with endometrial cancer [25]. These observations collectively support the notion that age represents an important clinical determinant of oncological outcomes and should be considered in prognostic assessment and treatment planning.

In our study, positive surgical margins were identified as an independent adverse prognostic factor for both PFS and OS. Surgical margin positivity may reflect residual tumor burden and microscopic disease dissemination, thereby increasing the likelihood of recurrence and cancer-related mortality. Notably, all 13 patients with positive surgical margins had FIGO 2023 stage III or IV, aggressive-histology tumors and had received adjuvant chemotherapy; this association should therefore be interpreted with caution, as it likely reflects advanced disease and greater tumor burden rather than an independent biological determinant. Previous studies have demonstrated that residual disease following primary cytoreductive surgery in advanced-stage endometrial cancer is associated with significantly poorer survival outcomes [26]. Together, these findings highlight the critical importance of optimal surgical resection and suggest that surgical quality represents a key prognostic determinant independent of FIGO stage.

Although the small number of downstaged patients (*n* = 4) limits statistical interpretation, their survival outcomes were comparable to those of early-stage patients. These findings suggest that downstaged patients exhibit a clinical course consistent with truly low-risk disease behavior and support the ability of the FIGO 2023 system to more accurately reflect tumor biology.

It is also noteworthy that stage migration, although significantly associated with survival in Kaplan–Meier analyses, did not emerge as an independent predictor in the multivariable Cox model. This finding is likely attributable to collinearity between stage migration and its constituent pathological drivers—namely LVSI, histological subtype, and molecular classification—which were independently retained in the final model. Accordingly, stage migration as a composite variable may reflect the cumulative effect of these underlying risk factors rather than representing an independent biological entity. Nonetheless, when FIGO 2023 stage group was modeled separately from these constituent factors and adjusted for age and surgical margin status, it retained a strong and independent association with both PFS and OS, with significantly higher risks observed for stage groups II–IV compared with stage group I (Supplementary Table S4). This indicates that the prognostic information of the revised system is preserved when it is analyzed as an integrated stage variable rather than decomposed into its individual components. Thus, the survival differences associated with stage migration should be interpreted as a manifestation of these underlying pathological drivers rather than as evidence that reclassification per se confers independent prognostic information.

Several criticisms of the FIGO 2023 staging system have been raised, particularly regarding the increased number of substages and the resulting complexity in routine clinical practice [27]. Nevertheless, our findings demonstrate that this complexity is accompanied by clinically meaningful separation in survival outcomes and results in a clinically relevant reclassification. The revised system nonetheless has intrinsic limitations: its reliance on molecular classification introduces interpretive challenges, including multiple-classifier tumors, incomplete concordance between immunohistochemistry and sequencing, and dependence on POLE sequencing, which is not universally available; these factors may limit its reproducibility and global applicability. In addition, the requirement for molecular testing and detailed substaging poses practical and financial barriers in resource-limited settings, where universal p53 and MMR immunohistochemistry and POLE sequencing may not be feasible, potentially limiting equitable implementation of FIGO 2023. Therefore, the improvement in prognostic accuracy justifies the increased level of detail and supports the notion that FIGO 2023 provides a staging approach that more accurately characterizes the biological behavior of the disease.

This study has several strengths that enhance its contribution to the existing literature. First, the use of real-world clinical data from two independent centers improves the external validity of the findings. Second, all patients were systematically restaged from FIGO 2009 to FIGO 2023, allowing a direct comparison of the prognostic implications of the two staging systems within the same cohort. Third, survival outcomes were evaluated using Kaplan–Meier analyses and multivariable regression models, enabling a robust assessment of independent prognostic factors. In addition, despite its retrospective design, the relatively high rate of molecular evaluation represents a notable strength of our study. In our cohort, 81.4% of patients were analyzed for p53 status and 78.7% for MMR status. Considering particularly the rates of p53 and MMR assessment, our study provides a robust molecularly characterized dataset compared with many previously published retrospective series. Nonetheless, the molecular profile of our cohort merits comment. The proportion of p53abn tumors (26.1%) was higher, and that of POLEmut tumors (0.3%) substantially lower, than the approximately 10–15% and 5–10% reported in unselected populations. This most likely reflects the tertiary-referral nature of the two participating centers—where aggressive, high-grade tumors are over-represented—together with the restriction of POLE sequencing to 68 patients (23.4%), with only one positive case (1.5%), rather than a true biological difference in our population. As a consequence, the prognostically favorable POLEmut subgroup could not be meaningfully evaluated, and the enrichment of p53abn tumors may have contributed to the observed stage-migration and survival patterns.

This study has several limitations that should be acknowledged. First, the retrospective design introduces inherent selection biases and limits causal inference. Second, the median follow-up was relatively short (29.2 months), and the limited number of events precluded estimation of median PFS and OS; this constrains the interpretability of survival estimates and warrants cautious extrapolation of long-term outcomes. Third, molecular profiling was incomplete across all patients. Molecular subtype could not be assigned in 24.4% of patients (*n* = 71). These patients were retained in the multivariable models as a separate ‘unknown’ category rather than excluded, thereby preserving statistical power; nonetheless, the incomplete molecular characterization limits the strength of the conclusions that can be drawn regarding the prognostic role of the molecular subtypes. Moreover, the number of events (67 progressions and 35 deaths) was modest relative to the number of candidate covariates, raising the possibility of model overfitting; to mitigate this, the composite stage-migration variable was removed and the multivariable models were kept as parsimonious as possible, and the wide confidence intervals for some estimates should be interpreted with caution. In particular, POLE sequencing was performed in only 68 patients (23.4%) and was positive in a single case (0.3% of the cohort, 1.5% of those sequenced), which substantially limited our ability to evaluate the prognostic impact of this molecularly defined subgroup and to fully assess downstaging driven by POLE mutation. Because only a single POLEmut case was identified, our conclusions cannot be extrapolated to this subgroup, and the true extent of POLE-driven downstaging is likely underestimated. Fourth, the number of downstaged patients was very small (*n* = 4), rendering any survival comparison in this group insufficiently powered and requiring cautious interpretation. Fifth, our study did not include a direct head-to-head comparison of the discriminative performance of the FIGO 2009 and FIGO 2023 systems using metrics such as the concordance index (C-index) or Akaike information criterion (AIC); future studies incorporating such analyses would further substantiate the prognostic superiority of the revised system. Finally, the single-country setting may limit generalizability to populations with different molecular testing practices or pathological reporting standards.

Our findings suggest that the FIGO 2023 staging system may improve prognostic stratification and facilitate selection of follow-up strategies and treatment modalities. Our study demonstrates that the FIGO 2023 staging system may provide more precise risk stratification, particularly in early-stage endometrial cancer, and reveals a clear survival disadvantage among patients who are upstaged. We emphasize, however, that these observations reflect improved prognostic separation rather than formally proven statistical superiority; a head-to-head comparison of discriminative ability (e.g., Harrell’s C-index or AIC) is required before FIGO 2023 can be declared superior to FIGO 2009. The integration of histopathological features and molecular biomarkers into the staging system also reflects the broader shift toward biologically informed and risk-adapted management strategies in endometrial cancer [11,12]. By incorporating tumor biology into the staging framework, the FIGO 2023 system may facilitate more individualized clinical decision-making and improve the alignment between staging and treatment planning. Future prospective multicenter studies are warranted to further clarify the prognostic and predictive value of this system and its integration into treatment algorithms.

## 5. Conclusions

In this study, histopathological features, particularly age, LVSI and surgical margin positivity, were identified as major determinants of prognosis. Restaging according to the FIGO 2023 system resulted in stage migration in approximately one-third of patients, and the upstaged group exhibited significantly inferior survival outcomes. In contrast, survival among downstaged cases was comparable to that of low-stage patients, suggesting that the new staging system more precisely characterizes the biological behavior of the disease. Importantly, in multivariable analysis, the prognostic signal attributed to stage migration was largely explained by its constituent pathological factors, including LVSI, histological subtype, molecular classification, and surgical margin status, rather than by reclassification itself. In conclusion, the integration of molecular and pathological parameters into staging indicates that the FIGO 2023 system represents not merely a more detailed staging system, but a clinically meaningful prognostic model that may inform risk stratification. However, because molecular characterization was incomplete and the impact of treatment was not directly analyzed, its implications for molecularly guided personalization and therapeutic decision-making remain to be confirmed in prospective studies.

## Figures and Tables

**Table 1:** Stage shifts between 2009 and 2023 FIGO staging systems.

FIGO 2023		FIGO 2009									
		IA	IB	II	IIIA	IIIB	IIIC1	IIIC2	IVA	IVB	
		*n* = 131	*n* = 53	*n* = 12	*n* = 14	*n* = 3	*n* = 24	*n* = 20	*n* = 3	*n* = 31	%
IAmPOLEmut	*n* = 1	-	-	**1(↓)**	-	-	-	-	-	-	0.3%
IA1	*n* = 23	23	-	-	-	-	-	-	-	-	7.9%
IA2	*n* = 63	63	-	-	-	-	-	-	-	-	21.6%
IA3	*n* = 3	-	-	-	**3(↓)**	-	-	-	-	-	1.0%
IB	*n* = 28	-	28	-	-		-	-	-	-	9.6%
IC	*n* = 4	**4(↑)**	-	-	-	-	-	-	-	-	1.4%
IIA	*n* = 3	-	-	3	-	-	-	-	-	-	1.0%
IIB	*n* = 11	**5(↑)**	**6(↑)**	-	-	-	-	-	-	-	3.8%
IIC	*n* = 50	**24(↑)**	**18(↑)**	8	-	-	-	-	-	-	17.2%
IICmp53abn	*n* = 13	**12(↑)**	**1(↑)**	-	-	-	-	-	-	-	4.5%
IIIA1	*n* = 9	-	-	-	9	-	-	-	-	-	3.1%
IIIA2	*n* = 1	-	-	-	1	-	-	-	-	-	0.3%
IIIB1	*n* = 2	-	-	-	-	2	-	-	-	-	0.7%
IIIB2	*n* = 2	-	-	-	**1(↑)**	1	-	-	-	-	0.7%
IIIC1	*n* = 24	-	-	-	-	-	24	-	-	-	8.2%
IIIC2	*n* = 20	-	-	-	-	-	-	20	-	-	6.9%
IVA	*n* = 3	-	-	-	-	-	-	-	3	-	1.0%
IVB	*n* = 13	-	-	-	-	-	-	-	-	13	4.5%
IVC	*n* = 18	-	-	-	-	-	-	-	-	**18(↑)**	6.2%
	**%**	45.0%	18.2%	4.1%	4.8%	1.0%	8.2%	6.9%	1.0%	10.7%	

**Abbreviations:** FIGO, International Federation of Gynecology and Obstetrics; p53abn, p53 abnormal; POLEmut, POLE mutated; (↑), Upstage (*n* = 89; 30.6%); (↓), Downstage (*n* = 4; 1.4%). Bold values indicate the number of patients whose stage changed after reclassification. Percentages are rounded to one decimal place and may not sum exactly.

**Table 2:** Univariate and multivariate analysis for progression-free survival.

Features		Median PFS (Months)	Univariate *p* Value	Multivariate *p* Value	HR (95% CI)
**Age (Years)**		NR	**0.001**	**0.004**	**2.37 (1.31–4.29)**
	<60	NR			
	≥60	NR			
**Stage Migration**		NR	**0.001**	**-**	**-**
**LVSI**		NR	**0.001**	**0.022**	**1.45 (1.06–1.99)**
**Myometrial Invasion**		NR	**0.001**	0.656	1.10 (0.72–1.68)
	Absent	NR			
	<50%	NR			
	≥50%	79.6			
**Histological Subtypes**		NR	**0.001**	0.716	0.95 (0.72–1.25)
**Histological Types**		NR	**0.001**	0.080	0.51 (0.24–1.09)
	Aggressive	41.2			
	Non-aggressive	NR			
**Surgical Margin**		NR	**0.001**	**0.001**	**2.27 (1.58–3.27)**
	Negative	NR			
	Positive	12.4			
**p53**		NR	**0.001**	0.758	0.94 (0.61–1.43)
	Wild-type	NR			
	Mutant	23.4			
**MMR**		NR	**0.001**	0.475	0.89 (0.65–1.23)
	Proficient	91.7			
	Deficient	NR			
**NSMP**		NR	**0.001**	0.145	0.33 (0.08–1.46)
	Yes	NR			
	No	91.7			
**ER**		NR	**0.001**	0.838	0.96 (0.68–1.38)
	Negative	22.7			
	Positive	91.7			
**PR**		NR	**0.001**	0.616	1.08 (0.79–1.48)
	Negative	29			
	Positive	91.7			

**Abbreviations:** HR, Hazard Ratio; CI, confidence interval; LVSI, lymphovascular space invasion; MMR, Mismatch Repair; NSMP, No Specific Molecular Profile; ER, estrogen receptor; PR, progesterone receptor; NR, not reached. Bold values indicate statistical significance (*p* < 0.05). Reference categories: age <60 years, LVSI absent, myometrial invasion absent, non-aggressive histology, surgical margin negative, p53 wild-type, MMR proficient, non-NSMP, and negative ER/PR. All covariates were analyzed as categorical variables. Missing values were retained as a separate ‘unknown’ category and were not imputed, so all patients were retained in the models. Multilevel variables (LVSI, myometrial invasion, and histological subtype) were entered into the Cox models as single ordinal terms; the reported hazard ratio therefore represents the effect per one-category increment, and separate category-specific hazard ratios were not estimated, although the reference category is indicated for each variable. The final multivariable PFS model included 290 patients and 66 progression events (one patient who did not undergo surgery could not be assigned a survival interval and was not included).

**Table 3:** Univariate and multivariate analysis for overall survival.

Features		Median OS (Months)	Univariate *p* Value	Multivariate *p* Value	HR (95% CI)
**Age (years)**		NR	**0.001**	**0.014**	**3.70 (1.31–10.46)**
	<60	NR			
	≥60	NR			
**Stage Migration**		NR	**0.001**	-	-
**LVSI**		NR	**0.001**	0.963	0.99 (0.63–1.56)
**Myometrial Invasion**		NR	**0.001**	0.503	1.24 (0.66–2.32)
	Absent	NR			
	<50%	NR			
	≥50%	97.3			
**Histological Subtypes**		NR	**0.001**	0.173	1.22 (0.92–1.63)
**Histological Types**		NR	**0.001**	0.287	0.51 (0.14–1.77)
	Aggressive	97.3			
	Non-aggressive	NR			
**Surgical Margin**		NR	**0.001**	**0.001**	**2.56 (1.48–4.42)**
	Negative	NR			
	Positive	25.5			
**p53**		NR	**0.001**	0.116	0.61 (0.33–1.13)
	Wild-type	NR			
	Mutant	43.8			
**MMR**		NR	**0.004**	0.889	1.03 (0.65–1.64)
	Proficient	NR			
	Deficient	NR			
**NSMP**		NR	0.089	0.749	1.30 (0.26–6.51)
	Yes	NR			
	No	NR			
**ER**		NR	**0.003**	0.800	1.07 (0.65–1.76)
	Negative	43.8			
	Positive	NR			
**PR**		NR	**0.001**	0.719	1.08 (0.70–1.69)
	Negative	43.8			
	Positive	NR			

**Abbreviations:** HR, Hazard Ratio; CI, confidence interval; LVSI, lymphovascular space invasion; MMR, Mismatch Repair; NSMP, No Specific Molecular Profile; ER, estrogen receptor; PR, progesterone receptor; NR, not reached. Bold values indicate statistical significance (*p* < 0.05). Reference categories: age <60 years, LVSI absent, myometrial invasion absent, non-aggressive histology, surgical margin negative, p53 wild-type, MMR proficient, non-NSMP, and negative ER/PR. All covariates were analyzed as categorical variables. Missing values were retained as a separate ‘unknown’ category and were not imputed, so all patients were retained in the models. Multilevel variables (LVSI, myometrial invasion, and histological subtype) were entered into the Cox models as single ordinal terms; the reported hazard ratio therefore represents the effect per one-category increment, and separate category-specific hazard ratios were not estimated, although the reference category is indicated for each variable. The final multivariable OS model included 280 patients and 34 deaths (the same patient without surgery, together with ten patients censored before the earliest event, did not contribute to the risk set).

## Data Availability

The data presented in this study are available on request from the corresponding author due to privacy/ethical restrictions.

## Supplementary Materials

The following supporting information can be downloaded at https://www.mdpi.com/article/10.3390/jcm15166326/s1: Table S1: Description of the 2009 FIGO staging system; Table S2: Description of the 2023 FIGO staging system; Table S3: Clinical and pathological characteristics of the study cohort; Table S4: Adjusted multivariable Cox regression using FIGO 2023 stage group as a categorical variable. Figure S1: Study flow diagram showing patient selection, exclusions, available molecular classification, and the final analytical cohort.
