# Supplementary material for: Prognostic Significance and Clinical Implications of the New FIGO 2023 Staging System in Patients with Endometrial Cancer
Source: J Clin Med. 2026 Aug 16;15(16):6326. doi: 10.3390/jcm15166326 (PMC13513586; doi:10.3390/jcm15166326)
Supplement: Supplementary file 1 [file jcm-15-06326-s001.zip › Supplementary_Tables.pdf]

Table S1. Description of the 2009 FIGO staging system.

Stage			Description
Stage I			Tumor confined to the corpus uteri
	IA		No or less than half myometrial invasion
	IB		Invasion equal to or more than half of the myometrium
Stage II			Tumor invades cervical stroma but does not extend beyond the uterus
Stage III			Local and/or regional spread of the tumor
	IIIA		Tumor invades the serosa of the corpus uteri and/or adnexa
	IIIB		Vaginal and/or parametrial involvement
	IIIC		Metastases to pelvic and/or para-aortic lymph nodes
		IIIC1	Positive pelvic nodes
		IIIC2	Positive para-aortic lymph nodes with or without positive pelvic lymph nodes
Stage IV			Tumor invades bladder and/or bowel mucosa, and/or distant metastases
	IVA		Tumor invasion of bladder and/or bowel mucosa
	IVB		Distant metastases, including intra-abdominal metastases and/or inguinal lymph nodes

Abbreviations: FIGO, International Federation of Gynecology and Obstetrics

Table S2. Description of the 2023 FIGO staging system.

Stage			Description
Stage I			Confined to the uterine corpus and ovary
	IA		Disease limited to the endometrium or non-aggressive histological type
	IAmPOLEmut		POLEmut endometrial carcinoma, confined to the uterine corpus or with cervical extension, regardless of LVSI or histological type
		IA1	Non-aggressive histological type limited to an endometrial polyp or confined to the endometrium
		IA2	Non-aggressive histological types involving less than half of the myometrium with no or focal LVSI
		IA3	Low-grade endometrioid carcinomas limited to the uterus and ovary
	IB		Non-aggressive histological types with invasion of half or more of the myometrium and with no or focal LVSI
	IC		Aggressive histological types limited to a polyp or confined to the endometrium
Stage II			Invasion of cervical stroma without extrauterine extension or with substantial LVSI or aggressive histological types with myometrial invasion
	IIA		Invasion of the cervical stroma of non-aggressive histological types
	IIB		Substantial LVSI of non-aggressive histological types
	IIC		Aggressive histological types with any myometrial involvement
	IICmp53abn		p53abn endometrial carcinoma confined to the uterine corpus with any myometrial invasion, with or without cervical invasion, and regardless of the degree of LVSI or histological type
Stage III			Local and/or regional spread of the tumor of any histological subtype
	IIIA		Invasion of uterine serosa, adnexa, or both by direct extension or metastasis
		IIIA1	Spread to ovary or fallopian tube (except when meeting stage IA3 criteria)
		IIIA2	Involvement of uterine subserosa or spread through the uterine serosa
	IIIB		Metastasis or direct spread to the vagina and/or to the parametria or pelvic peritoneum
		IIIB1	Metastasis or direct spread to the vagina and/or the parametria
		IIIB2	Metastasis to the pelvic peritoneum
	IIIC		Metastasis to the pelvic or para-aortic lymph nodes or both
		IIIC1	Metastasis to the pelvic lymph nodes
		IIIC1i	Micrometastasis
		IIIC1ii	Macrometastasis
		IIIC2	Metastasis to para-aortic lymph nodes up to the renal vessels, with or without metastasis to the pelvic lymph nodes
		IIIC2i	Micrometastasis
		IIIC2ii	Macrometastasis
Stage IV			Spread to the bladder mucosa and/or intestinal mucosa and/or distant metastasis
	IVA		Invasion of the bladder mucosa and/or bowel (intestinal) mucosa
	IVB		Abdominal peritoneal metastasis beyond the pelvis
	IVC		Distant metastasis, including metastasis to any extra- or intra-abdominal lymph nodes above the renal vessels, lungs, liver, brain, or bone

Abbreviations: FIGO, International Federation of Gynecology and Obstetrics; LVSI, lymphovascular space invasion; p53abn, p53 Abnormal; POLEmut, POLE mutated

Table S3. Clinical and pathological characteristics of the study cohort.

		Number of Patients (N)	Percentage (%)
**Age (years)**			
	<60	128	44
	≥60	163	56
**Family History of Endometrial Cancer**			
	Yes	15	5.2
	No	276	94.8
**Family History of Other Cancer**			
	Yes	66	22.7
	No	225	77.3
**Surgery**			
	Yes	290	99.7
	No	1	0.3
**Type of Surgery**			
	Laparotomy	132	45.5
	Laparoscopic	148	51
	Robotic	6	2.1
	Unknown	4	1.4
**pT**			
	T1A	148	50.9
	T1B	69	23.7
	T2	22	7.6
	T3A	33	11.3
	T3B	10	3.4
	T4	7	2.4
	TX	2	0.7
**pN**			
	N0	224	77
	N1	28	9.6
	N2	35	12
	NX	4	1.4
**pM**			
	M0	256	88
	M1	33	11.3
	MX	2	0.7
**LVSI**			
	Absent	171	58.8
	Focal	15	5.2
	Substantial	88	30.2
	Unknown	17	5.8
**Myometrial Invasion**			
	Absent	43	14.8
	<50%	122	41.9
	≥50%	113	38.8
	Unknown	13	4.5
**Histological Subtype**			
	Endometrioid	201	69.1
	Serous	57	19.6
	Carcinosarcoma	13	4.5
	Clear cell	6	2.1
	Mixed histology	8	2.7
	Mesonephric-like	3	1
	Undifferentiated carcinoma	3	1
**Histological Type**			
	Aggressive	123	42.3
	Non-aggressive	168	57.7
**Surgical Margin**			
	Positive	13	4.5
	Negative	256	88.3
	Unknown	21	7.2
**Molecular Subtypes**			
	p53abn mutant	76	26.1
	POLEmut	1	0.3
	dMMR	81	27.8
	NSMP	62	21.3
	Unknown / unclassified	71	24.4
**Stage Migration**			
	Upstaging	89	30.6
	Downstaging	4	1.4
	No change	198	68
**Neoadjuvant Chemotherapy**			
	Yes	5	1.7

	No	286	98.3
**Adjuvant Chemotherapy**			
	Yes	134	46
	No	157	54
**Brachytherapy**			
	Yes	113	38.8
	No	178	61.2
**Radiotherapy**			
	Yes	109	37.5
	No	182	62.5
**ER**			
	Positive	176	60.5
	Negative	29	10
	Unknown	86	29.5
**PR**			
	Positive	143	49.1
	Negative	32	11
	Unknown	116	39.9
**Progression**			
	Absent	224	77
	Present	67	23

Abbreviations: ER, estrogen receptor; LVSI, lymphovascular space invasion; p53abn, p53 abnormal; POLEmut, POLE mutated; dMMR, Mismatch Repair–Deficient; NSMP, No Specific Molecular Profile; PR, progesterone receptor

Table S4. Adjusted multivariable Cox regression using FIGO 2023 stage group as a categorical variable.

FIGO 2023 stage group	PFS HR (95% CI)	p	OS HR (95% CI)	p
I	1.00 (reference)	—	1.00 (reference)	—
II	9.26 (3.17–27.04)	<0.001	15.97 (2.05–124.30)	0.008
III	8.61 (2.88–25.73)	<0.001	14.34 (1.76–117.13)	0.013
IV	25.05 (8.30–75.63)	<0.001	49.53 (6.29–389.74)	<0.001

Note: The wide confidence intervals reflect the small number of events in the stage I reference group.

Abbreviations: CI, confidence interval; FIGO, International Federation of Gynecology and Obstetrics; HR, hazard ratio; OS, overall survival; PFS, progression-free survival.
